# *E2F4* Promotes the Proliferation of Hepatocellular Carcinoma Cells through Upregulation of *CDCA3*

**DOI:** 10.7150/jca.53708

**Published:** 2021-06-22

**Authors:** Junye Liu, Lulu Xia, Shilei Wang, Xuefei Cai, Xiaoli Wu, Chunhong Zou, Baoju Shan, Miao Luo, Deqiang Wang

**Affiliations:** 1 Key Laboratory of Molecular Biology for Infectious Diseases, Ministry of Education, Department of Infectious Diseases, Institute for Viral Hepatitis, Chongqing Medical University, Chongqing, China.; 2 Department of Dermatology and Cosmetology, Chongqing Hospital of Traditional Chinese Medicine, Chongqing, China.; 3 College of Laboratory Medicine, Chongqing Medical University, Chongqing, China.; 4 Pediatric Research Institute; Ministry of Education Key Laboratory of Child Development and Disorders; National Clinical Research Center for Child Health and Disorders (Chongqing); China International Science and Technology Cooperation base of Child development and Critical Disorders; Children's Hospital of Chongqing Medical University, Chongqing, China.; 5 Chongqing Key Laboratory of Pediatrics, Children's Hospital of Chongqing Medical University, Chongqing, China.; 6 Department of Clinical Laboratory, Yubei District People's Hospital, Chongqing, China.

**Keywords:** hepatocellular carcinoma, E2F4, CDCA3, promoter

## Abstract

Liver cancer, the second most commonly diagnosed cancer, is associated with high mortality rates. *E2F4* is a member of the E2F transcription factor family. There are limited studies on the role of *E2F4* in hepatocellular carcinoma (HCC). In this study, the expression of *E2F4* in HCC tissue samples and cell lines was analyzed using quantitative real-time polymerase chain reaction. *E2F4* expression positively correlated with tumor size in patients with HCC. Additionally, *E2F4* expression was greater in HCC cells than in normal LO2 cells. Furthermore, overexpression of *E2F4* significantly enhanced the proliferation, migration, and invasion of HCC cells. The results of a luciferase assay revealed that *E2F4* upregulated the expression of *CDCA3* by binding to its promoter region (1863'-ACGCGCGAGAATG-1875') and consequently promoted proliferation and cell cycle progression of HCC cells. Taken together, these results demonstrated that E2F4 might play a vital role in HCC progression and could serve as a potential biomarker for the diagnosis and as a therapeutic target of HCC.

## Introduction

The incidence of liver cancer, which is the second most commonly diagnosed cancer overall, is higher among men than women. In developed countries, liver cancer is the fifth leading cause of cancer-related deaths among men. The incidence of liver cancer is also high in developing countries. Oftentimes, no symptoms are presented during early stage disease. Hence, liver cancer tends to be diagnosed at an advanced stage, especially in developing countries. The incidence of liver cancer is lowest in South Asia, Northern Europe, Central Europe, and Eastern Europe and highest in East Asia, Southeast Asia, North Africa, and West Africa. Globally, the most common primary liver cancer is hepatocellular carcinoma (HCC), which accounts for 75%-85% of all primary liver cancer cases 1-3.

The E2F transcription factor family is involved in cell proliferation, apoptosis, differentiation, aging, DNA damage response, and DNA repair 4-9. This family was first identified in 1987 as transcription factors required to activate the E2 adenovirus promoter. Currently, nine members of the family have been identified: E2F1, E2F2, E2F3a, E2F3b, E2F4, E2F5, E2F6, E2F7, and E2F8, which are located on different chromosomes. The genes encoding E2F family transcription factors are highly homologous and encode highly conserved DNA binding, dimerization, and retinoblastoma (RB) protein-binding domains 10. This family can be divided activator (E2F1, E2F2, and E2F3a) and repressor (E2F3b, E2F4, E2F5, E2F6, E2F7, and E2F8) E2F transcription factors based on their structure and function.

Studies have shown that E2F mRNA expression levels are related to different cancer stages and pathological grades of HCC patients, and E2F4 protein is highly expressed in advanced liver cancer 11. E2F4 is the most abundantly expressed E2F transcription factor. Additionally, E2F4, which accounts for the majority of E2F activity, is involved in various cellular functions. E2F4 has previously been reported to function as a transcriptional repressor. However, recent studies have reported transcriptional activation functions as well 12,13.

The role of *E2F4* in HCC pathogenesis remains unclear. Hence, this study aimed to elucidate the role of *E2F4* in HCC. The expression of *E2F4* in clinical HCC tissues and adjacent non-tumorous tissues, as well as in HCC and normal liver cell lines, was examined using quantitative real-time polymerase chain reaction (qRT-PCR). The findings of this study indicated that E2F4 might promote HCC carcinogenesis and might function as a candidate biomarker for diagnosis and as a therapeutic target.

## Materials and methods

### Clinical samples

HCC and adjacent tissues were collected from 40 patients undergoing thoracic surgery in the Department of Hepatobiliary Surgery, the First Affiliated Hospital of Chongqing Medical University, from January 2018 to January 2019. The samples were collected from 33 male and seven female patients. The patients were randomly selected, all showed primary HCC, and had not been treated with radio- or chemotherapy before surgery. Based on the tumor, node, metastasis (TNM) staging, 11, 7, and 22 cases were classified as stage I, stage II, and stage III, respectively. The tissues were immediately placed in RNA preservation solution in liquid nitrogen after surgical removal. The study protocol was approved by the Research Ethics Committee of the First Affiliated Hospital of Chongqing Medical University, and written informed consent was obtained from each patient.

### Cell lines and cell culturing

Five human HCC cell lines (Huh7, Hep3B, HepG2, Bel-7402, and SK-HEP-1) and one modified normal liver cell line (LO2) were purchased from the American Type Culture Collection (Manassas, VA, USA). HepG2 cells were cultured in minimal essential medium (MEM) supplemented with 10% fetal bovine serum (FBS). The other cells were cultured in Dulbecco's Modified Eagle Medium (DMEM) supplemented with 10% FBS. All cells were cultured in a cell incubator at 37 °C and 5% CO_2_. The pcDNA3.1 plasmid and the adenoviral vector encoding control short interfering RNA (siRNA) and red fluorescence protein (RFP) were obtained from the Infectious Disease Laboratory of Chongqing Medical University. The pcDNA3.1-E2F4 overexpression plasmid and the adenoviral vector encoding si-E2F4 (Ad-siE2F4) were constructed by our group and verified by sequencing. Plasmid transfection was performed using Lipofectamine 3000, according to the manufacturer's instructions. The virus titers used for cell infection were >10× 11.

### Materials and reagents

The following materials and reagents were used in this study: DMEM, MEM, and trypsin (Gibco, Waltham, MA, USA); FBS (Natocor Industria Biologica, Cordoba, Argentina); Lipofectamine 3000 kit (Invitrogen, Carlsbad, CA, USA); reverse transcription kit and polyvinyl difluoride (PVDF) membranes (Roche, Basel, Switzerland); SYBR green qPCR master mix (MCE, Monmouth Junction, NJ, USA); bicinchoninic acid (BCA) protein quantitative analysis kit (ThermoFisher Scientific, Waltham, MA, USA); dual-luciferase kit (Promega, Madison, WI, USA); transwell chambers, 96-well plates, cell culture dishes (Corning, Corning, NY, USA); enhanced chemiluminescence (ECL) chromogenic solution (Bio-Rad, Hercules, CA, USA); 30% acrylamide-bis-acrylamide solution (LABTIDE, China); human anti-glyceraldehyde-3-phosphate dehydrogenase (GAPDH) antibody, goat anti-rabbit secondary antibody, goat anti-mouse secondary antibody, anti-CDCA3 antibody (Proteintech, Wuhan, China); and anti-E2F4 monoclonal antibody (Santa Cruz Biotechnology, Dallas, TX, USA).

### RNA extraction, reverse transcription, and qRT-PCR

Total RNA was extracted from tissues and cells using TRIzol following the manufacturer's instructions. cDNA was synthesized using 1 µg of the isolated RNA as a template. The synthesized cDNA was diluted using double distilled water (1:9) and stored at -20 °C until use. qRT-PCR analyses were performed in triplicate. The relative expression levels of *E2F4* were calculated using the 2^-ΔΔCt^ method. The primers used for qRT-PCR were listed in Table S1.

### Cell proliferation, migration, and invasion assays

Cells were transfected with pcDNA3.1-E2F4 or Ad-siE2F4 for 24 h. The cells were then resuspended and counted using a cow-abalone counting plate. The proliferation of cells (2 × 10^3^/well) seeded in a 96-well plate was examined. When the cells adhered, a long-term dynamic cell imaging system was used to analyze the number of cells in each well and set as an initial value. Cells were counted once every 24 h, and the growth rate was calculated. A colony formation assay was performed using a six-well plate. Cells (500 cells/well) were cultured for one week and stained with crystal violet, and the colonies were counted. To perform the wound-healing assay, 4 × 10^4^ cells/well were plated in a 96-well plate. When the cells adhered to the wall and the density reached at least 90%, the monolayer was scratched to introduce a wound gap. A long-term dynamic cell imaging system was used to capture images of the cells once every 12 h to analyze wound healing. Cell invasion assays were performed using transwell chambers. The upper chamber was coated with Matrigel (1:10). Cells (4 × 10^4^) were resuspended in serum-free DMEM and seeded into the upper chamber. DMEM supplemented with 10% FBS was added to the lower chambers and the cells were incubated for 24 h. Transwell inserts were then removed and the cells were washed three times with phosphate-buffered saline. The cells were fixed in 4% paraformaldehyde at room temperature for 30 min, stained with crystal violet for 10 min, and counted using an optical microscope.

### Dual-luciferase reporter assay

Huh7 cells were co-transfected with pCDNA3.1-E2F4, Renilla Fluorescein TK, pGL3-CDCA3, pGL3-CDCA3-Δ1, pGL3-CDCA3-Δ2, or pGL3-CDCA3-Δ3 constructs. At 48 h or 72 h post-transfection, cells were harvested and lysed. The fluorescence intensity of the cell lysate was measured using a GloMax fluorescence detector (Promega).

### Statistical analysis

All statistical analyses were performed using SPSS 22.0. Data were expressed as the mean ± standard deviation from three independent experiments. The means between two groups were analyzed using Student's *t*-test, while those between multiple groups were analyzed using one-way analysis of variance (ANOVA). The correlation between *E2F4* expression and the clinicopathological features of patients was analyzed using the chi-square (χ^2^) test. Differences were considered significant at *P* <0.05.

## Results

### Correlation between E2F4 expression and clinicopathological factors

**Figure 1:**
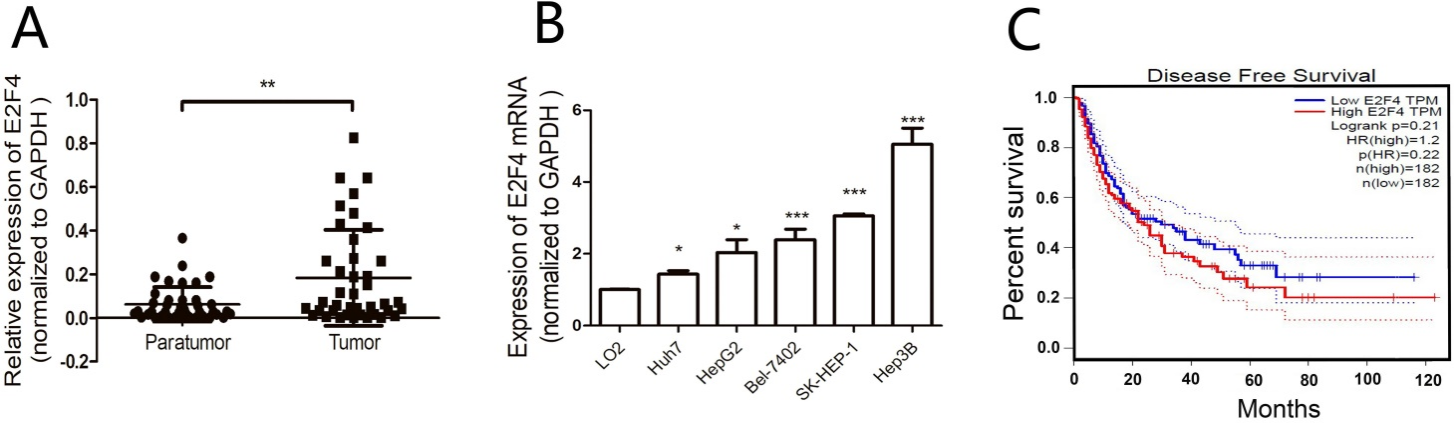
Expression of *E2F4* in the hepatocellular carcinoma (HCC) samples and the correlation with clinicopathological features.** A.** Expression of *E2F4* in HCC and adjacent non-tumorous tissues was examined using quantitative real-time polymerase chain reaction (qRT-PCR).** B.** Expression of *E2F4* in Huh7, SK-HEP-1, Hep3B, HepG2, and Bel-7402 cells. The immortalized normal liver cell line (LO2) was used as a control. **C.** The gene expression profiling interaction analysis (GEPIA; http://gepia.cancer-pku.cn/index.html) database was used to evaluate the correlation between *E2F4* expression and the survival rate of patients with HCC.

The mRNA levels of *E2F4* in matched HCC and adjacent non-tumorous tissues from 40 HCC patients were analyzed using qRT-PCR. The clinical tissue samples were divided into *E2F4*-high (21 cases) and *E2F4*-low groups (19 cases) based on median *E2F4* expression (Table 1). Analysis of the correlation between *E2F4* expression and clinicopathological features revealed that most patients with tumor sizes > 5 cm (16/24) exhibited upregulated *E2F4* expression. This suggests that the expression of *E2F4* is positively correlated with tumor size. The absolute expression of *E2F4* in the adjacent non-tumorous tissues (0.062 ± 0.0125) was significantly lower than the tumorous tissues (0.183 ± 0.035) (Fig. 1A). Additionally, *E2F4* mRNA levels were examined in HCC (Huh7, SK-HEP-1, Hep3B, HepG2, Bel-7402) and normal liver (LO2) cell lines. Among the HCC cell lines, Huh7 and Hep3B exhibited the lowest and highest *E2F4* expression, respectively. *E2F4* expression in all HCC cell lines was higher than LO2 cells (Fig. 1B). These results suggest that *E2F4* functions as an oncogene in liver cancer tissues. Survival analysis using the gene expression profiling interaction analysis (GEPIA; http://gepia.cancer-pku.cn/index.html) database revealed that the survival rate of HCC patients in the *E2F4*-high cohort was lower than that of patients in the *E2F4*-low cohort (Fig. 1C), which was consistent with the findings of Huang et al. 11,14. This suggests that *E2F4* is involved in HCC development.

### Effect of E2F4 on the HCC cell proliferation, migration, and invasion

**Figure 2:**
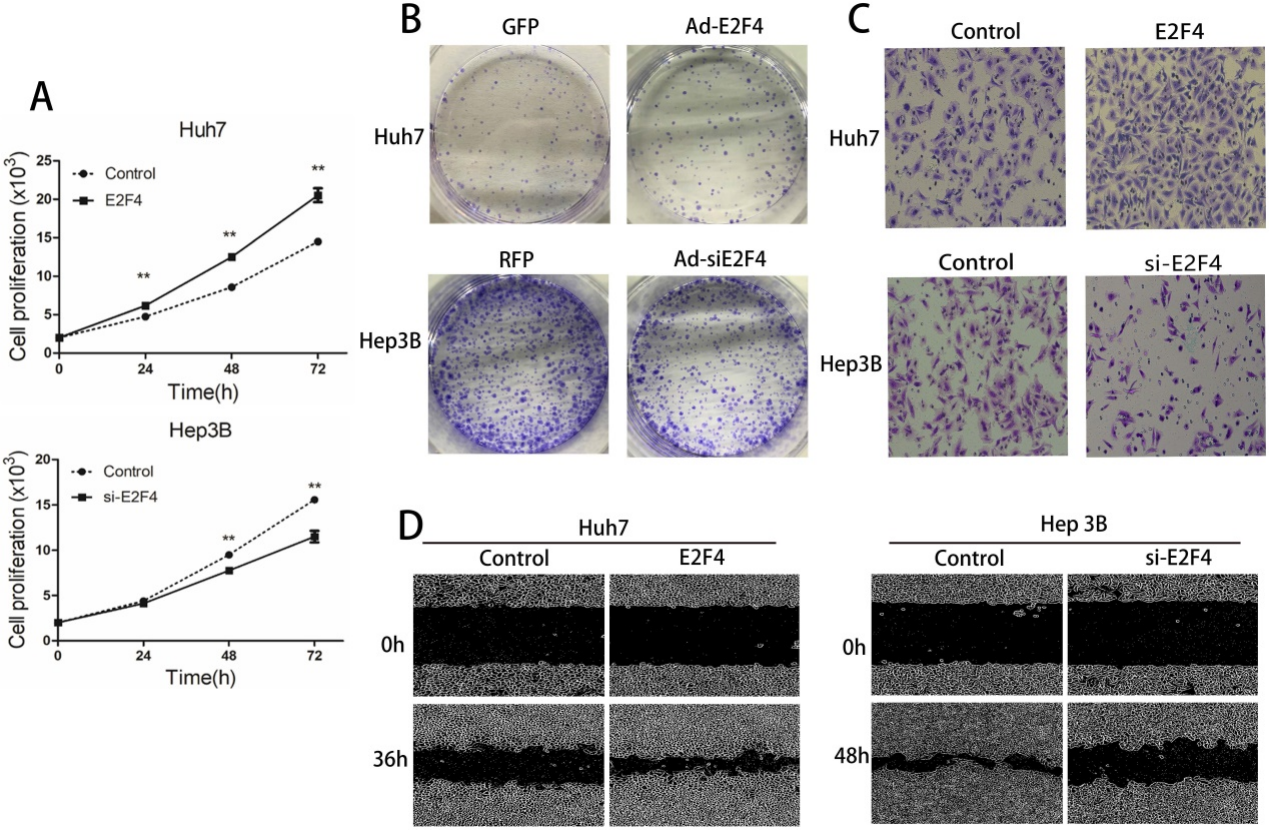
Effect of *E2F4* overexpression or knockdown on hepatocellular carcinoma (HCC) proliferation, migration, and invasion. *E2F4* was overexpressed in Huh7 and knocked down in Hep3B cells.** A.** Results of the cell proliferation assay. The transfected cells were seeded and imaged using a long-term dynamic cell imaging system once every 24 h and the growth rate was calculated.** B.** Results of the colony formation assay. The transfected cells were cultured for one week. The cells were stained to examine the growth of colonies. **C.** Results of the transwell assay. The cells that passed through the membrane were stained and counted using a microscope (Magnification: 200×).** D.** Results of the wound-healing assay. The cell monolayer was scratched, and the migration of cells into the scratch area was monitored once every 12 h using the long-term dynamic cell imaging system.

The proliferation, migration, and invasion of Ad-si*E2F4*-transfected and Ad-*E2F4*-transfected HCC cells were examined next. As shown in Fig. 2A, the proliferation of Ad-*E2F4*-transfected Huh7 cells was greater than the control cells. The proliferation of Hep3B cells was significantly decreased upon transfection with Ad-si*E2F4*. The results of the colony formation assay revealed that the Ad-*E2F4*-transfected Huh7 cells exhibited an increased number of colonies (Fig. 2B), whereas the Ad-si*E2F4*-transfected Hep3B cells exhibited a significantly decreased number of colonies. In the wound-healing assay, the migration rate of Ad-*E2F4*-transfected Huh7 cells significantly increased (Fig. 2C), while for Ad-si*E2F4*-transfected Hep3B cells, it significantly decreased. The results of the transwell assay (Fig. 2D) revealed that the invasion of Ad-*E2F4*-transfected Huh7 cells was markedly higher than the control cells. In contrast, the invasion of Ad-si*E2F4*-transfected Hep3B cells was markedly decreased. These findings further confirmed that *E2F4* could enhance the proliferation, migration, and invasion of HCC cells 15.

### E2F4 promotes the progression of HCC cell cycle

E2F family transcription factors mediated several cellular functions, including apoptosis and proliferation. E2F-mediated enhancement of cell proliferation could be caused by either inhibition of apoptosis or enhancement of proliferation 16.

**Figure 3:**
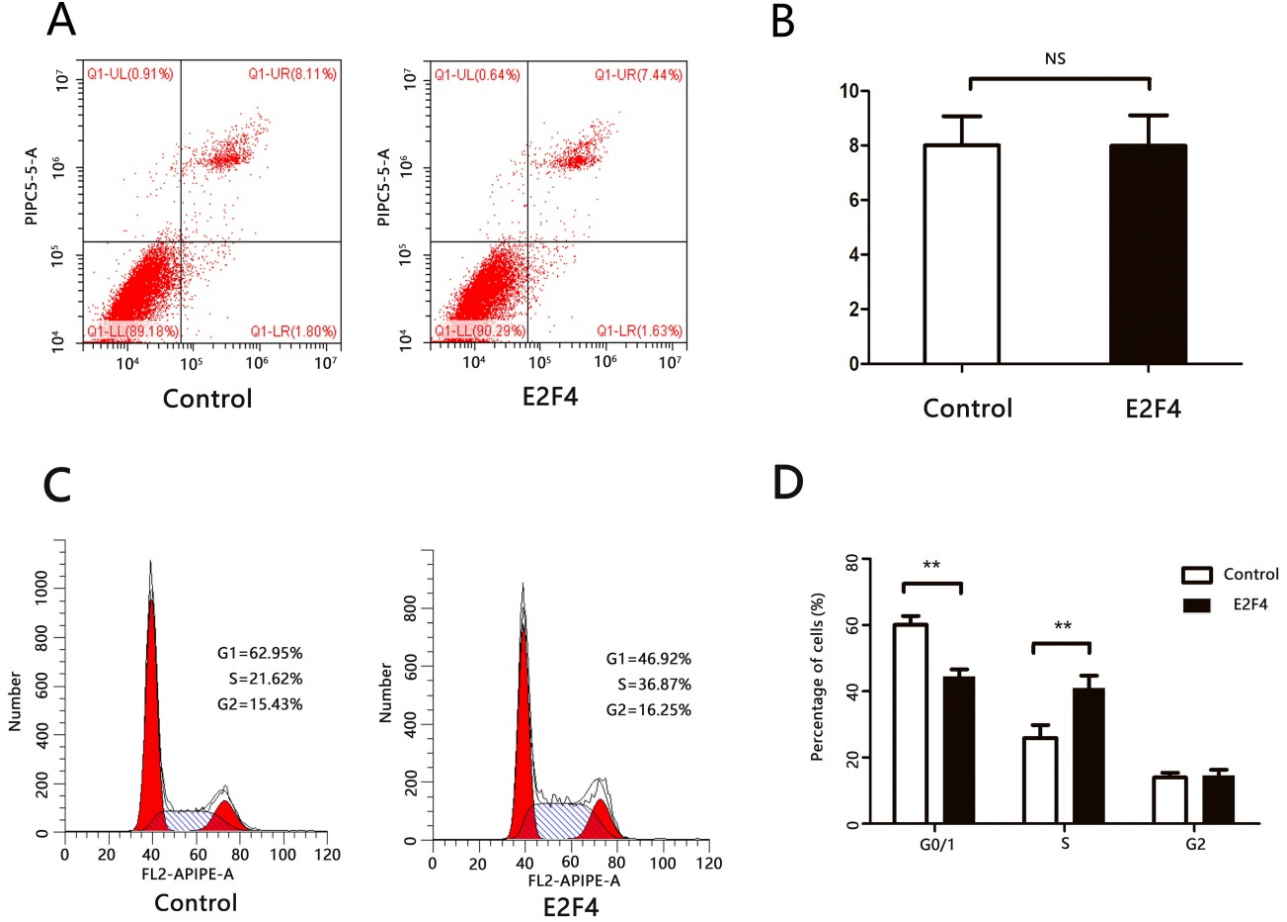
*E2F4* can promote cell cycle progression of hepatocellular carcinoma (HCC) cells. Huh7 cells were transfected with pCDNA3.1 or pCDNA3.1-E2F4. At 48 h post-transfection, the cells were analyzed via flow cytometry. **A.** Effect of *E2F4* overexpression on apoptosis. **B.** Quantification of the number of late apoptotic cells in the pCDNA3.1-E2F4- and pCDNA3.1-transfected groups (upper right quadrant). **C.** Effect of *E2F4* overexpression on the cell cycle.** D.** Quantification of the proportion of cells in the G0/1, S, and G2 phases of the cell cycle in the pCDNA3.1-E2F4- and pCDNA3.1-transfected groups.

Huh7 cells transfected with either pcDNA3.1 or pcDNA3.1-E2F4 and subjected to flow cytometry analysis to examine apoptosis and cell cycle markers at 48 h or 72 h post-transfection. There was no significant difference in the number of cells at the late apoptotic (upper right quadrant) stage between the pcDNA3.1- and pcDNA3.1-*E2F4*-transfected groups (Figs. 3A and 3B). This suggests that *E2F4* does not promote apoptosis in HCC cells. The pcDNA3.1-*E2F4*-transfected group exhibited an increased number of cells in the S-phase (Figs. 3C and 3D), indicating that *E2F4* promotes the transition from G0/1 to S-phase and contributes to cell cycle progression.

### CDCA3 is a target gene for E2F4

**Figure 4:**
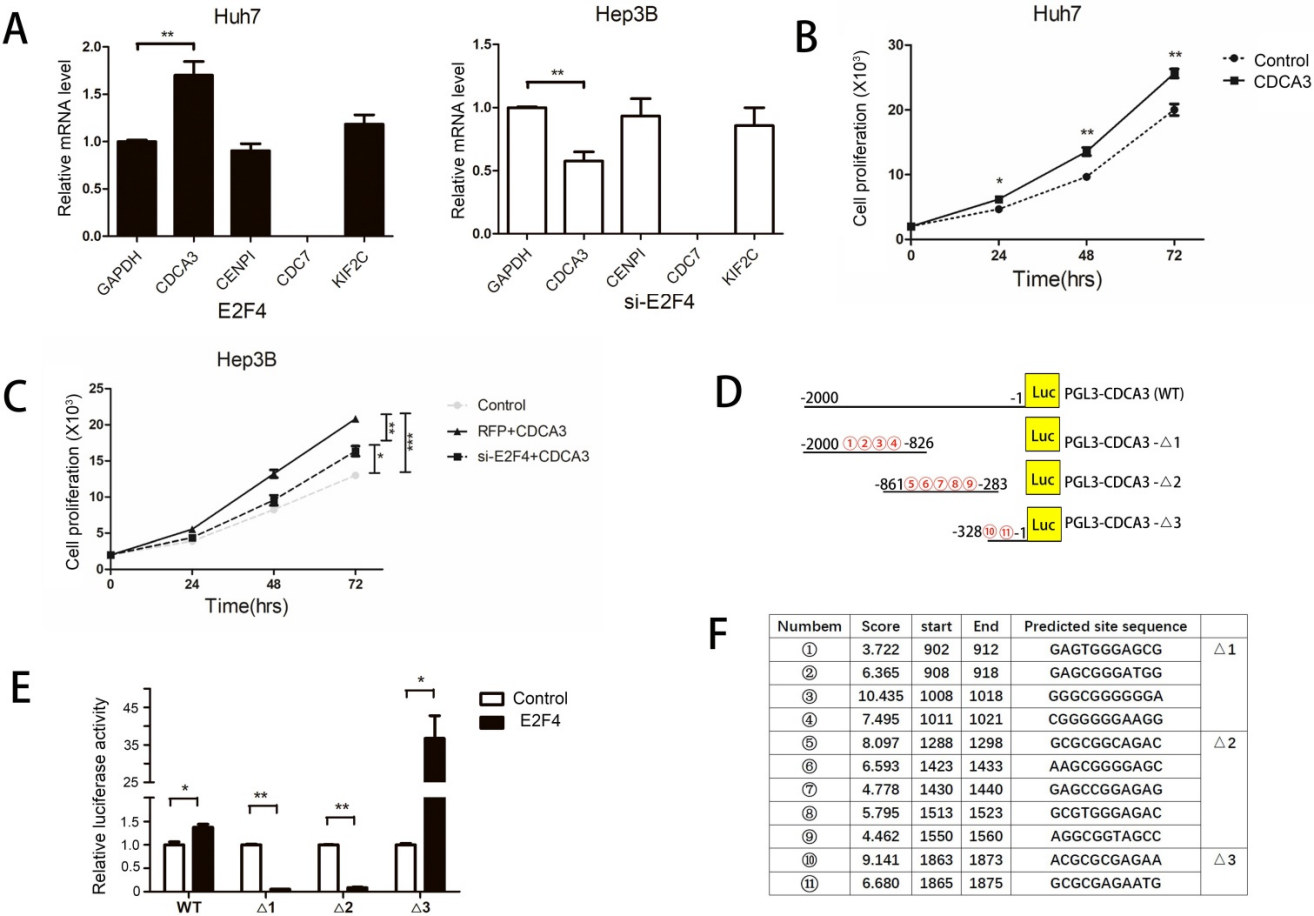
Identification of E2F4 target genes. **A.** *E2F4* was overexpressed in Huh7 cells and knocked down in the Hep3B cells. The expression of *CDCA3*, *CENPI*, *CDC7*, and *KIF2C* was examined using quantitative real-time polymerase chain reaction (qRT-PCR). **B-C.** The proliferation of Huh7 or Hep3B cells exhibiting overexpression or knockdown of *E2F4*. **D-F.** Schematic representation of the full-length and truncated *CDCA3* promoters. 1 to 11 represent the predicted E2F4 binding sites. Results of the dual-luciferase reporter assay. Huh7 cells were transfected with *CDCA3* full-length promoter [pGL3-CDCA3 (WT)] or three truncated *CDCA3* promoter (pGL3-CDCA3-Δ1, pGL3-CDCA3-Δ2, and pGL3-CDCA3-Δ3) constructs. Luciferase activity was examined at 48 h post-transfection.

*E2F4* regulates the expression of various genes by binding to promoters 17. As E2F4 promotes the progression of the HCC cell cycle, we hypothesized that it modulated the expression of cell cycle regulator genes. Previous studies have reported various target genes of *E2F4* using chromatin immunoprecipitation (ChIP) analysis. Among these target genes, *CDCA3*, *CENPI*, *CDC7*, and *KIF2C* were involved in the regulation of the cell cycle 12,13. The effect of *E2F4* overexpression or knockdown on the expression of these four genes in HCC cells was examined using qRT-PCR. The expression of *CDC7* was not detected in both Huh7 and Hep3B cells and expression of *CENPI* and *KIF2C* were unaffected by either overexpression of knockdown of *E2F4*. However, the expression of *CDCA3* was positively correlated with that of *E2F4* (Fig. 4A). CDCA3, a cell division cycle protein, promoted the proliferation and cell cycle progression of colorectal and gastric cancer cells 14. The proliferation of CDCA3-transfected cells significantly increased and was significantly inhibited upon co-transfection with siE2F4 (Figs. 4B and 4C). This indicates that *E2F4* regulates HCC cell cycle through *CDCA3*.

The E2F4 binding sites located in the *CDCA3* 5'-untranslated region (UTR) comprise a 2000-bp sequence upstream of the transcription start site, which were analyzed using the UCSC database. According to the JASPAR database, 11 candidate sequences in the *CDCA3* promoter region were predicted to serve as potential E2F4 binding sites (Figs. 4D and 4F). Therefore, we constructed a vector encoding the full-length *CDCA3* promoter (pGL3-CDCA3; pGL3-Basic as the vector) as well as vectors encoding three *CDCA3* truncated promoter constructs containing three central high-scoring sequences (with relatively strong binding potential) (pGL3-CDCA3-Δ1, pGL3-CDCA3-Δ2, and pGL3-CDCA3-Δ3) (Fig. 4D). A dual-luciferase reporter assay was performed by co-transfecting Huh7 cells with *E2F4* and pGL3-CDCA3, pGL3-CDCA3-Δ1, pGL3-CDCA3-Δ2, or pGL3-CDCA3-Δ3. Huh7 cells co-transfected with *E2F4* and PGL3-CDCA3-Δ3 exhibited the highest luciferase activity (Fig. 4E). Two candidate sequences in the pGL3-CDCA3-Δ3 construct, 1863-ACGCGCGAGAA-1873 and 1865-GCGCGAGAATG-1875, were located at the same site. This indicates that E2F4 binds to this 13-bp sequence (1863-ACGCGCGAGAATG-1875) in the *CDCA3* promoter.

## Discussion

The E2F transcription factor family is involved in various cellular functions 15,16. Previous studies have reported that E2F1 mediates carcinogenesis in various cancers. However, recent studies have also demonstrated that E2F1 is positively correlated with the tumor cell apoptosis index in cell carcinoma. Thus, E2F1 is a pro-apoptotic regulator that can also activate the tumor suppressor p53 17,18. Some studies have reported that E2F4 can arrest the cell cycle at G0 19,20. E2F4 is involved in the carcinogenesis of skin tumors, gastrointestinal tumors, and prostate cancer 21,22. This study reported, for the first time, that E2F4 couldpromote the carcinogenesis of HCC through the upregulation of CDCA3, a cell cycle-related protein.

In the G0 phase, activator E2Fs often form a complex with RB family members (including RB, p107, and p130). The cyclin/CDK complex phosphorylates the RB protein upon stimulation, resulting in the release of E2F. Free E2F can induce the transcription of cell cycle-related proteins and consequently promote cell cycle progression 4,5,23-26. E2F4, a member of the E2F transcription factor family, may dissociate from phosphorylated RB and upregulate *CDCA3* expression by binding to its promoter region (1863-ACGCGCGAGAATG-1875). The upregulated expression of *CDCA3* promotes the proliferation and cell cycle progression of HCC cells. However, further studies are required to elucidate the underlying mechanisms.

In this study, we analyzed the expression of *E2F4* in HCC tissue samples and cell lines. *E2F4* expression positively correlated with tumor size. The expression of *E2F4* in HCC cell lines was higher than in LO2 cells. *E2F4* overexpression enhanced the proliferation, migration, and invasion of HCC cells. The results of the luciferase reporter assay revealed that E2F4 upregulates *CDCA3* expression by binding to its promoter region (1863-ACGCGCGAGAATG-1875) and consequently promotes the proliferation and cell cycle progression of HCC cells.

## Supplementary Material

Supplementary table S1.

## Figures and Tables

**Table 1:** Relationship between *E2F4* expression and clinicopathological characteristics in 40 patients with HCC

Factor	E2F4 expression		χ^2^ value	*P* value
	High (N=21)	Low (N=19)		
**Age**			0.316	0.574
<40 years	3	4		
≤40 years	18	15		
**Gender**			1.948	0.163
Male	19	14		
Female	2	5		
**HBsAg**			0.025	0.874
+	17	15		
-	4	4		
**HBV DNA**			0.351	0.554
+	13	10		
-	8	9		
**AFP**			2.03	0.154
<400 μg/L	15	17		
≥400 μg/L	6	2		
**ALT**			0.422	0.516
<40 U/L	10	11		
≥40 U/L	11	8		
**AST**			0.902	0.342
<40 U/L	12	8		
≥40 U/L	9	11		
**Tumor size**			4.829	0.028
<5 cm	5	11		
≥5 cm	16	8		
**TNM stage**			0.819	0.366
I	3	8		
II	4	3		
III	14	8		

TNM, tumor, node, metastasis; HBsAg, hepatitis B surface antigen; HBV, hepatitis B virus; ALT, alanine aminotransferase; AST, aspartate aminotransferase; AFP, alpha-fetoprotein.

## Abbreviations

HCC: hepatocellular carcinoma
TNM: tumor, node, metastasis
qRT-PCR: quantitative real-time polymerase chain reaction
