# Supplementary material for: E2F4 Promotes the Proliferation of Hepatocellular Carcinoma Cells through Upregulation of CDCA3
Source: J Cancer. 2021 Jun 22;12(17):5173–80. doi: 10.7150/jca.53708 (PMC8317516; doi:10.7150/jca.53708)
Supplement: Supplementary file 1 — Supplementary table S1. [file jcav12p5173s1.pdf]

Supplementary table S1: The list of primers used for qRT-PCR

Primers	Sequences （5’ - 3’）
GAPDH-F	GTATGACAACGAATTTGGCTACAG
GAPDH-R	TGAGGGTCTCTCTCTTCCTCTTGT
E2F4-F	TGCCACCACCTGAAGATTT
E2F4-R	GGAGTGAGCTGAGGACTATTTG
